# Sustainable Alternatives in Multilayer Packaging: Storage Stability of Pudding Powder Under Accelerated Storage Conditions

**DOI:** 10.3390/foods14223806

**Published:** 2025-11-07

**Authors:** Can Türksever, Banu Koç, Ozlem Kizilirmak Esmer

**Affiliations:** 1 Graduate School of Natural and Applied Sciences, Ege University, İzmir 35040, Türkiye; 2 Gastronomy and Culinary Arts Department, Faculty of Tourism, Gaziantep University, Gaziantep 27310, Türkiye; banukoc@gantep.edu.tr; 3 Department of Food Engineering, Faculty of Engineering, Ege University, İzmir 35040, Türkiye

**Keywords:** sustainable packaging, accelerated storage testing, pudding powder, multi-layered films, metallized film, aluminum oxide coating, mono-layered film

## Abstract

Multilayer packaging materials are extensively used in food packaging, particularly for powdered products. In alignment with sustainable development goals, packaging design should aim to minimize material usage while maintaining the protective properties necessary to preserve food quality and safety, thereby reducing environmental impact. A key strategy is to simplify multilayer structures to enhance recyclability. This study aims to evaluate the potential of sustainable alternative packaging materials with reduced metal and plastic content and improved recyclability for pudding powder packaging, as substitutes for conventional films. Four packaging structures were tested: a conventional three-layer laminate (polyethylene terephthalate (PET)/aluminum foil (Al-foil)/low-density polyethylene (LDPE)), two two-layer structures (AlO_x_-coated PET/LDPE and Al-coated PET/LDPE), and a monolayer metallized biaxially oriented polypropylene (MetBOPP). Samples were stored under accelerated conditions (38 °C and 90% relative humidity) for 180 days, and changes in moisture content, water activity, caking degree, glass transition temperature, color, and sensory attributes were monitored. The experimental data were examined for their agreement with various sorption models by creating adsorption isotherms. The acceptable storage period was estimated using the constants calculated from these models. Statistically significant differences (*p* < 0.05) were observed among the packaging types, primarily associated with their water vapor permeability, affecting moisture content, water activity, caking degree, and color stability. In terms of moisture content, water activity, and caking degree, the conventional PET/Al-foil/LDPE (Polyethylene terephthalate/Aluminum foil/Low density polyethylene) structure demonstrated the best performance, followed by PET.AlO_x_/LDPE (AlO_x_-coated Polyethylene terephthalate/Low density polyethylene), MPET/LDPE (Metallized polyethylene terephthalate/Low density polyethylene), and MBOPP (Metallized biaxially oriented polypropylene), respectively. The sensory analysis scores followed the same ranking; however, all samples maintained scores above the threshold value of 3 throughout the storage period, indicating that they remained acceptable. Caking degree increased moderately (from 0.61% to 0.89%) and was negatively correlated with appearance scores (R^2^ = −0.89, *p* < 0.01). Despite slight darkening (Browning Index increased from 18.16 to 20.37), sensory scores for appearance, odor, and taste remained above the acceptable threshold (score > 3.0). Based on the WVTR values of the packaging materials and the application of the GAB model, the estimated shelf lives were 800.32 days for PET/Al-foil/LDPE, 577.92 days for PET.AlO_x_/LDPE, 407.58 days for MPET/LDPE, and 229.26 days for MBOPP. In conclusion, the longest shelf life was achieved with PET/Al-foil/LDPE, and it was observed that as the WVTR of the packaging materials increased, the shelf life of the cocoa-based pudding powder decreased; PET.AlO_x_/LDPE and MPET/LDPE could be considered for medium-term storage (up to about 1–1.5 years), while MBOPP appeared suitable only for shorter durations (6–8 months).

## 1. Introduction

Growing concerns about environmental pollution, particularly in industrialized countries, have reinforced the perception that packaging is a waste. As a result, efforts to raise global consumer awareness of the environmental impact of packaging waste have increased, and sustainable development has gained importance. In this context, packaging contributes to sustainable development by ensuring consumer health and safety, while reducing product losses and food waste and extending shelf life [1], thereby supporting both social and environmental sustainability [2]. In definitions of sustainable packaging provided by various organizations, the importance of packaging design is consistently emphasized, particularly in terms of minimizing the use of energy and materials. Accordingly, to reduce environmental impact and enhance recyclability, packaging design should be adapted to the product’s characteristics, aiming to decrease material thickness, weight, and the number of layers wherever possible [3,4,5,6,7,8,9].

Due to their ease of processing, low cost, durability, and lightweight, plastics are extensively used across various sectors, with the packaging industry alone accounting for 44% of global plastic consumption [10]. However, if not recycled, plastic materials can remain in nature for hundreds of years without decomposing and pose significant environmental problems such as groundwater contamination, soil degradation, and marine ecosystem threats, affecting both human health and ecological balance [11]. Therefore, to minimize the environmental impact and improve the recyclability of petroleum-derived plastic packaging materials, it is important to design the packaging according to the characteristics of the product and to optimize the barrier properties and the number of layers in multi-layer packaging as much as possible.

Multilayer packaging, which combines the superior properties of distinct materials, typically paper, plastic, and aluminum has significant applications in the food packaging sector due to its excellent barrier performance against water vapor and gases such as oxygen, carbon dioxide, and aromatic compounds [12]. However, the main drawback of multilayer packaging is its poor recyclability, resulting in a very low recycling rate [13]. This limitation arises because multilayer structures pose significant challenges to current recycling systems and conflict with the principles of the circular economy, mainly due to the use of dissimilar materials in each layer, the large differences in their processing properties, the absence of detection systems for multilayer identification, and the lack of dedicated collection infrastructure [14]. Especially in flexible materials, the thin nature and low bulk density of flexible packaging pose technical challenges in recycling processes, rendering these materials economically inefficient and consequently less favored by recycling companies [15]. Therefore, the packaging waste of multilayer flexible packaging materials is often collected and incinerated in landfills [16], contributing to environmental pollution and resource depletion [17].

Aluminum is one of the most commonly used materials in multilayer combinations owing to its nearly perfect barrier properties against gasses, and liquids, and its perfect light barrier [18,19]. Aluminum can be incorporated into packaging materials through lamination and coating processes in multilayer packaging. In the lamination, the plastic materials are often laminated with an aluminum foil and the resulting laminated material has a considerably low water vapor transmission rate (WVTR) and oxygen transmission rate (OTR). But in the coating, aluminum is evaporated within a vacuum chamber, and the resulting metal vapor is deposited onto a cold film [20]. The aluminum-coated plastics, which are also known as metallized plastics, are economically and environmentally more advantageous than plastics laminated with aluminum foil, and they also have good barrier properties, although not as much as plastics laminated with aluminum foil [16]. The aluminum consumption is much less in metallized films than when aluminum is used as a layer in multilayer packaging due to the extremely thin layer of metallized coating which is only several ten nm. However, the thickness of the aluminum layer in flexible laminate is typically 7–9 µm [21]. Another alternative method of using aluminum to reduce aluminum consumption in multilayer packaging is coating plastic films with aluminum oxide (AlO_x_) at a nanometer level. Various deposition techniques can be used to form such inorganic coatings on polymeric films. Among these, the vacuum deposition methods have gained significant attention for creating transparent barrier layers. In these processes, aluminum is evaporated under vacuum, and a controlled amount of oxygen is introduced into the vapor phase. The aluminum atoms react with oxygen to form a thin, transparent aluminum oxide layer on the substrate surface, which provides excellent barrier properties when deposition parameters are properly optimized. When polyethylene terephthalate (PET) is employed as the substrate, this reactive evaporation approach yields stable and reproducible barrier performance, while maintaining transparency, microwave compatibility, and lightweight structure [22,23]. When applied on polyolefin films, they can reduce the OTR by nearly 90% [24]. Therefore, such coated films can be regarded as mono-material structures. AlO_x_-coated materials, particularly on BOPP and PET films, have shown excellent barrier performance and are gaining attention for food packaging applications [22,23,24,25,26]. Nevertheless, it has been reported that such coatings may develop cracks, particularly when applied on flexible substrates, which can compromise their barrier performance. To improve mechanical stability and barrier durability, these layers are often incorporated between polymer layers in multilayer structures [13]. Moreover, during thermal processing, the metal oxide layer of these films may develop pinholes and cracks, which further increase oxygen and moisture permeabilities [27,28]. Despite these potential issues, Parhi et al. [28] demonstrated that AlO_x_-coated PET films can effectively extend the shelf life of low-acid foods sterilized by microwave-assisted thermal sterilization, performing better than Al-foil-based packaging materials. Similarly, Patel et al. [27] showed that double-layer AlO_x_-coated PET films provided a comparable shelf life for chicken pasta sterilized by microwave without compromising the films’ barrier properties, compared to Al-foil pouches.

In multilayer flexible packaging, minimizing the quantity of material used and the number of layers holds significant importance in enhancing the contribution of packaging to sustainable development [1]. Therefore, the choice of aluminum as a coating or a layer will have an impact on the recyclability of multilayer packaging [29]. In “A Global Recommendation for Circular Packaging Design” by WPO [7], it was stated that components made of metal, aluminum-containing materials (with a layer thickness > 5 µm) lead to difficulties in recycling as they interfere with sorting. Since metallized films and AlO_x_ coatings are extremely thin—typically accounting for less than 5% of the total substrate weight—they do not interfere with the recycling process. Given their extremely thin coatings, these films are regarded as a mono-material structure [22].

The packaging industry faces an increasing pressure to reduce the use of multilayer packaging. Therefore, in last decades, trends to mono-layer materials containing predominantly only one type of material have increased due to their easier processing in conventional recycling systems, particularly in Europe, with support from initiatives like the CEFLEX Consortium [30].

The shelf-life of food products is closely linked to the barrier properties of the packaging materials used. Most foods are sensitive to moisture loss or absorption, which is governed by the WVTR, to oxygen-induced degradation, such as oxidation reactions, which depend on the OTR, and to microbiological growth, which is influenced by both WVTR and OTR. To address these challenges, multilayer flexible packaging materials are commonly employed in packaging designs, due to their excellent barrier properties [13].

Powdered foods, owing to their low moisture content and water activity, have an extended shelf life. Their sensory shelf life is affected by quality issues such as flavor and aroma loss, crystallization, agglomeration and deterioration of flow characteristics during storage [31,32]. The adverse effects of humidity and oxygen in the packaging environment on the product quality of such goods are mitigated using packaging materials with high moisture and oxygen barrier properties [33].

Pudding powders are the products that can be prepared easily at home and offer a delicious flavor that can be consumed as a dessert or used as an ingredient in dessert recipes. They are commonly formulated without milk or cream to make them less expensive and gain higher storage stability [34,35]. Different types of pudding powders exist according to flavor and aroma, with cocoa, vanilla, and banana being common examples. Pudding powder is highly sensitive to moisture due to its composition, which typically includes hygroscopic ingredients such as starch and sucrose. These components readily absorb moisture from the environment, leading to a reduction in the glass transition temperature, surface plasticization, and ultimately caking and loss of product quality. Therefore, moisture control is a critical factor in ensuring the physical stability and shelf life of such powders. This makes pudding powder an appropriate and representative model product for evaluating the water vapor barrier properties of different packaging materials. Furthermore, pudding powder and similar dry dessert mixes are widely consumed due to their affordability and convenience. However, they are commonly sold in multilayer films with high barrier properties, such as PET, aluminum, LDPE; PP (polypropylene) and EVOH (ethylene vinyl alcohol) or metal cans can be used in the packaging of pudding powders [34,36]. While these provide effective moisture protection, they are not easily recyclable, raising environmental concerns. Given the high consumption volume of such products, improving the sustainability of their packaging is both relevant and necessary. Thus, the objective of this study goes beyond merely comparing the technical performance of alternative packaging materials—it also addresses the broader issue of transitioning toward more sustainable packaging solutions for widely used products.

The shelf life of foods generally varies depending on the physical, chemical, and microbiological changes that occur in the food. Water in foods is an important parameter for controlling the rate of spoilage. While spoilage in foods with a water activity of 0.7–1.0 is associated with microbial spoilage, spoilage in foods with a value between 0.4–0.7 is associated with browning reactions and lipid oxidation. Generally, foods with a water activity of around 0.3 have a low rate of all types of spoilage reactions [37,38]. The packaging design should be made according to the characteristics of the food in a way that will have less impact on the environment, and preserve the characteristics of the product [2,3,7,9]. In this context, powdered foods containing protein, sugar, and fat such as milk powder are typically prone to moisture absorption, agglomeration, and chemical deterioration reactions like the Maillard reactions and oxidation. Therefore, packaging with low oxygen and water vapor permeability is generally recommended [39]. However, since pudding powders mainly consist of sugar and starch, controlling the water vapor permeability of the packaging material alone may be sufficient to ensure product stability to prevent water absorption leading to clumping and stickiness of sugar and gelatinization of starch [40]. Physical deterioration reactions such as caking and clumping occur due to changes in moisture content, water activity, and glass transition temperature which are influenced by storage conditions and the water vapor permeability of the packaging material. Two factors must be taken into account when predicting the shelf life of delicate to moisture products such as pudding powder: (1) the movement of vaporized water inside the packaging, and (2) moisture gain of the product [41]. The rate of moisture transfer to the product depends on the packaging properties and storage conditions such as surface area, relative humidity (RH), temperature and water vapor permeability while moisture gain of the product is contingent on its moisture sorption behavior. Thus, determining the sorption isotherms of materials is essential; however, only a few studies have reported the moisture sorption isotherms of Palada payasam (rice flakes milk pudding) mix [42], and instant pudding powder mix [43].

In packaging studies, storage testing is typically conducted using two primary testing procedures: real-time tests and accelerated tests. In real-time testing, a product is stored under recommended conditions and monitored until it fails to meet specifications. This conventional method is time-consuming and costly because it estimates shelf life under normal day-to-day conditions. However, accelerated shelf-life testing (ASLT) is performed relatively quickly by estimating shelf life under extreme conditions, such as high temperature and humidity levels, which are different from normal storage conditions. Potter [44] indicated that accelerated storage conditions with high humidity and temperature, such as 38 °C and 90% RH can be used to rapidly enhance the understanding of the relationship between water vapor transfer and storage time. Accelerated storage conditions of 38 °C and 90% RH have been widely adopted in various studies concerning powdered food products, including tamarind pulp powder [45], honey powder [46], apple peel powder [47], aloe vera gel powder [48], coconut milk powder [49], mango soy-fortified yoghurt powder [50], and mango powder [51]. To the best of our knowledge, limited studies have been reported in the literature regarding the evaluation of storage stability of pudding powders.

This study aimed to evaluate the potential of using more recyclable and environmentally friendly packaging materials for cocoa pudding powders by reducing the number of layers in conventional multilayer structures and exploring the feasibility of mono-layer alternatives. The formulation of the powder pudding used in this study contains 14.9% low-fat cocoa powder, which contributes minimally to the overall fat content but can influence moisture interactions and storage stability, highlighting the importance of selecting appropriate packaging materials. While multilayer structures such as PET/Al-foil/LDPE are commonly used for packaging powdered food products due to their excellent barrier properties, this study also investigated the suitability of alternative two-layer materials employing Al coatings (MPET/LDPE) or AlO_x_ coatings (PET.AlO_x_/LDPE). Furthermore, the potential application of the mono-layer metallized biaxially oriented polypropylene (MBOPP film), despite its relatively higher WVTR and OTR, has been investigated as a more sustainable packaging solution for pudding powder. The storage conditions were 38 °C/90% RH. Throughout a six-month storage period, samples were analyzed at one-month intervals to assess changes in moisture content, water activity, color, caking degree, glass transition temperature, and sensory attributes. The adsorption kinetics of pudding powders were investigated at 38 °C.

## 2. Materials and Methods

### 2.1. Materials

The cocoa pudding powder formulized with sugar (55%), corn starch (30%), reduced-fat cocoa powder (14.9%), and salt (0.1%) was used. Four types of packaging materials were used for the packaging of pudding powder. One of these was a three-layered PET (Polyethylene Terephthalate, 12 µm)/Al-Foil (Aluminum foil, 9 µm)/LDPE (Low-Density Polyethylene, 60 µm), with a WVTR of 0.049 g/m^2^·day and an OTR of 0.026 cc/m^2^·day·atm, which is commonly used for powder pudding packaging. The other three alternative materials were PET.AlO_x_ (AlO_x_-coated PET, 12 µm)/LDPE (50 µm), MPET (Metallized Polyethylene Terephthalate, 12 µm)/LDPE (50 µm), and MBOPP (Metallized Biaxially Oriented Polypropylene, 30 µm). Their WVTRs were 0.190, 0.356, and 0.650 g/m^2^·day, respectively, and their OTRs were 0.130, 0.335, and 12.190 cc/m^2^·day·atm. The packaging materials are presented in Table 1.

### 2.2. Fitting of Sorption Models to Experimental Data

The adsorption isotherms were determined using the standard static, gravimetric method at a constant temperature of 38 °C [52,53]. The samples were dried in an oven at 60 °C for 4 h before testing. Ten salts were selected (LiCl, CH_3_COOK, MgCl_2_, K_2_CO_3_, Mg(NO_3_)_2_, NaBr, SrCl_2_, NaCl, NH_3_SO_4_, KCl) to cover a water activity range from 11 to 87%. The saturated salt solutions were prepared according to the recommendations of the COST 90 project [53,54]. Samples weighing 0.3 g (± 0.001 g) for adsorption, were placed in a tared sample dish and placed in jars containing the saturated salt solutions. Each jar contained two sample dishes for two parallel runs. The values for the relative humidity values of the saturated salt solutions at 38 °C were taken from Greenspan [55]. To prevent mold growth under experimental conditions of more than 50% relative humidity (RH), the containers filled with 5 mL toluene were placed in each sorption jar [53,54]. The sorption jars were placed in a temperature-controlled oven, and the subsequent adsorption experiments were carried out and repeated at constant temperature of 38 °C. The samples were allowed to equilibrate for a period of one and a half months, during which their weight was monitored until a stable weight (± 0.01 g) was reached.

A review of the existing literature concerning the depiction of sorption isotherms using mathematical models has revealed that sorption isotherms of foods can be described by several sorption models [56]. The criteria for selecting the most suitable sorption models are the degree of compatibility of the model with the experimental data and the simplicity of the proposed model. In the evaluation of experimental data, all equations in Table 2 were analyzed.

The model’s parameters were ascertained through non-linear regression analysis utilizing the curve fitting tools in MATLAB 2022a. This process involved minimizing the sum of squared errors based on the experimental sorption data. In order to assess the appropriateness of the considered equation, the mean % relative deviation (P) and the root % mean squared error (RMSE) values were considered as the basis.

(1) $$P\%=\frac{100}{N}\sum_{i=1}^{N}\frac{\left|M_{ei}-M_{ci}\right|}{M_{ei}}$$

(2) $$RMSE\%=\sqrt{\frac{1}{N}\sum_{i=1}^{N}{\left(\frac{\left|M_{ei}-M_{ci}\right|}{M_{ei}}\right)}^{2}\times 100}$$

### 2.3. Setting Up Accelerated Storage Conditions of Packaged Pudding Powder and Investigation of Quality Parameters

#### 2.3.1. Sample Preparation

The packaging materials were cut in 12.5 cm × 17 cm dimensions and prepared in the form of bags by using a heat sealing machine (PackTech, İzmir, Türkiye). Each of these bags were filled with 150 g of pudding powder. The sample preparation process was duplicated twice for each sample.

#### 2.3.2. Accelerated Storage

The sample groups were subjected to storage conditions of 90% RH at 38 °C for 180 days. To achieve the 90% RH environment, a saturated potassium chloride solution was employed.

#### 2.3.3. Water Vapor Transmission Rate

The water vapor transmission rate was determined in accordance with the ASTM F1249 method under the conditions of 38 °C and 90% RH. Two parallel analyses were conducted for each film sample, and the results were expressed as g/m^2^·day [63].

#### 2.3.4. Oxygen Transmission Rate

The oxygen transmission rate was determined in accordance with the ASTM D3985 method under the conditions of 23 °C and 0% RH. Two parallel analyses were conducted for each film sample, and the results were expressed as cc/m^2^·day·atm [64].

#### 2.3.5. Moisture Content

The moisture content of samples was gravimetrically determined. 1 g of sample was dried in an oven at 105 °C until reaching a constant weight. Three replications were conducted for each sample [65].

#### 2.3.6. Water Activity

The water activity was determined by a water activity meter (Testo AG 400, Lenzkirch, Germany) with a sensitivity of ±0.001. A sample weighing 2 g was placed in a stainless-steel container, and the system was considered to have reached equilibrium when the water activity value remained stable with a variation of less than 0.001 for 10 min. Three replications were conducted for each sample.

#### 2.3.7. Caking Degree

The degree of caking was determined by the gravimetric method as percentage by weight, as described by Goula and Adamopoulos [66]. It was quantified by subjecting the sample to an atmosphere with a RH of 75% which was generated through a saturated salt solution of sodium chloride at a controlled temperature of 20 ± 2 °C for 90 min. The powdered food product was dried and sieved through a 500 µm sieve, and the amount of sample retained on the sieve was weighed. The degree of caking was calculated using the following equation.

(3) $$Degree\;of\;Caking\;\left(\%\right)=\frac{b\times 100}{a}$$

a is the amount of dried powder (g), b is the amount of dried powder (g) retained in the sieve.

#### 2.3.8. Color

The color of samples was analyzed according to the Hunter L^*^, a^*^, and b^*^ color systems by Hunterlab ColorFlex colorimeter (Reston, VA, USA). Four replications were conducted for each sample. BI (browning index) values of the samples were calculated using Equations (3) and (4) [67].

BI = [100 (x − 0.31)]/0.172(4)

x = (a^*^ + 1.75 L^*^)/(5.645 L^*^ + a^*^ − 0.3012 b^*^) (5)

#### 2.3.9. Glass Transition Temperature

Differential scanning calorimetry (DSC Q2000, TA Instruments, Xew Castle, DE, USA) was used to determine the glass transition temperature of the samples. A sample weighing 3 µg was put into 20 µL aluminum DSC sample pans, and hermetically sealed. The analysis encompassed a temperature range from 0 to 200 °C, with the temperature being elevated at a rate of 20 °C per min. Dry nitrogen gas of a purity of 99.9% at a flow rate of 20 mL/min was used as the purifying gas. The thermal history of each sample was removed by performing an initial heating scan. The glass transition temperature (T_g_) was then determined from a second heating run, based on the midpoint of the change in heat capacity recorded during the DSC analysis.

#### 2.3.10. Sensory Evaluation

A scoring test [68] was applied for sensory analysis. In these tests, a group of nine trained panelists was employed. The criteria for evaluating the pudding powder were established in preliminary trials, and a sensory panel form was developed (Table S1). The analysis employed a quantitative scoring system, rating the appearance, smell, and taste of the pudding powder on a scale from 1 to 5, where 1 represented “very poor” and 5 indicated “excellent.” A score of 3 was set as the threshold for product acceptability.

#### 2.3.11. Acceptable Storage Period

The. acceptable storage period was calculated using the following equation [41]:

(6) $$\int t=\frac{w_{d}}{k\times A}\left(\int_{m_{i}}^{m_{f}}\frac{d_{m}}{\left(p_{0}-p_{i}\right)}\right)$$

where t is the storage time (days), w_d_ is the dry weight of pudding powder (g), m is the moisture content of powder on % dry basis, m_i_ is the initial moisture content (%, db) of the sample, m_f_ is the final moisture content (%, db), k is the water vapor permeability coefficient (g·m^2^·day^−1^·Pa^−1^), A is the area of the package (m^2^), p_0_ is the partial pressure of water vapor (Pa) of storage environment, and p_i_ is the partial pressure of water vapor (Pa) inside the package headspace related to the moisture content of the product determined by GAB model.

#### 2.3.12. Statistical Analysis

All measurements were performed in triplicate, and the replicates were used for statistical evaluation. A two-way analysis of variance (ANOVA) was conducted to assess the effects of storage time and packaging material on product quality parameters. Since a significant interaction between the two factors was observed (*p* < 0.05), simple effects were further examined. Specifically, one-way ANOVA was applied (i) within each packaging material to determine the differences among storage times, and (ii) within each storage time to determine the differences among packaging materials. Multiple comparisons were performed using Tukey’s HSD test to identify significant differences between groups. The significance level was set at *p* < 0.05 for all statistical tests. Replicates were treated as random effects in the model, and analyses were conducted using SPSS Statics 24.0 (SPSS Inc., Chicago, IL, USA). In addition, the quality properties were correlated, and correlation coefficients were calculated.

## 3. Results

### 3.1. Fitting of Sorption Models to Experimental Data

As with all other foods, the moisture content (MC) of the pudding powder is a decisive factor in determining the conditions for its processing, storage, and packaging. The relationship between the moisture content of food and the water activity of the medium at constant temperature and pressure is described by the moisture sorption isotherm (MSI) of foods. The equilibrium moisture content (EMC) of the samples in equilibrium in an environment with varying relative humidity and constant temperature increased with increasing relative humidity of the atmosphere. The influence of MC on the reactions taking place in the food is expressed by the water activity (a_w_); thus, it is possible to control the reactions and ensure the stability of the food [69]. The optimum MC of food corresponds to the capacity of the monolayer at which the intensity of the reactions taking place is the lowest [43]. By modelling the shelf life of powdered foods with low moisture content, the application of the sorption isotherms can save money and time without the need for long-term analyses [70]. Experimental methods are usually used to determine the sorption isotherms, and the most appropriate sorption models are estimated by fitting the experimental data to mathematical models from the literature [71].

**Figure 1:**
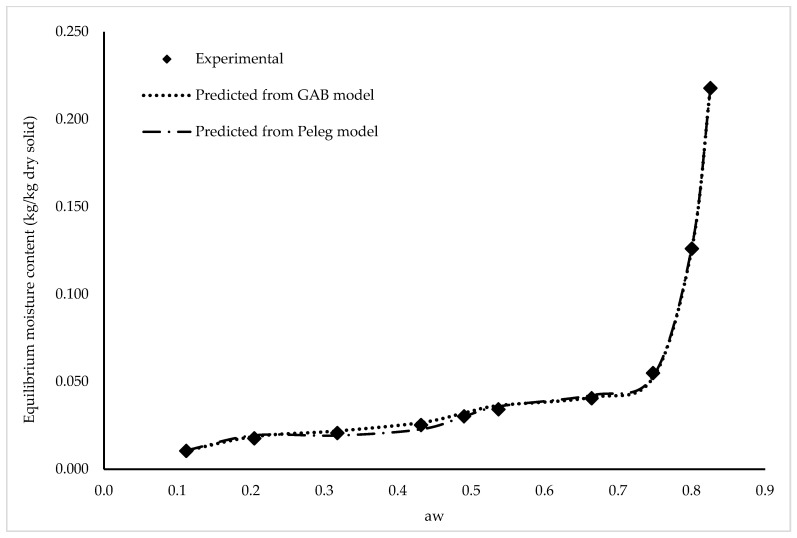
Experimental and predicted adsorption equilibrium moisture contents data for powder pudding at 38 °C using GAB and Peleg Models.

The isotherm of the moisture sorption of the pudding powder at 38 °C is shown in Figure 1. The EMC at each a_w_ is the average of two replicates, with the standard deviation for each test point (kg/kg dry solids) ranging from 0.037 to 1.118. The EMC of pudding powder increased with increasing a_w_. For pudding-like products, this behavior has also been observed in other studies [42,43]. Based on the BET classification, it was found that the isotherms have a typical J-shape belonging to the type ΙΙΙ isotherm, also called Flory-Huggins isotherm, which is characterized by absorbing modest amounts of water at low a_w_ values and larger quantities of water at high relative humidy. The type III isotherm (type J) is the sorption isotherm of pure crystalline solids such as sugars and salts or foods with high sugar content [60].

As shown from Figure 1, the graph of the adsorption isotherm shows that the moisture uptake at a_w_ ≤ 0.7 was very slow and uniform, followed by an accelerated steep increase. The moisture sorption isotherms of pudding powder showed a curvature at the point where the water activity value was 0.75. The reason for this curvature is the increase in the amount of water adsorbed by the product with the increase in a_w_ and, accordingly, the increase in the volume of moisture retained by the product and the upward curvature of the isotherm. It is assumed that this behavior is due to solid foods that are rich in soluble compounds such as sugar [72], and this point has been determined in the literature to be 0.8 of a_w_ for pure crystalline sugars [73]. The increase in moisture is very small, up to the point where the crystals commence dissolution in the absorbed water on the crystal surface [74]. Furthermore, in this study, as the water activity (a_w_) value exceeded 0.75, a more rapid increase in the equilibrium moisture content of pudding powder was observed. A similar phenomenon was observed in the research conducted by Ocieczek and Ruszkowska [43] with instant pudding and it was attributed to the more pronounced adsorption of water vapor and the occurrence of capillary condensation in samples above this water activity value (0.75).

The adsorption data of pudding powder were fitted to the mathematical equations GAB, BET, Halsey, Iglesias-Chirife, Henderson, and Peleg, which are commonly used for experimental sorption data of various foods, and the parameters obtained by regression analysis and the coefficients of determination R^2^, RMSE%, and P% are shown in Table 2. It was found that the model GAB developed by Guggenheim Anderson and de Boer for multilayer adsorption has three parameters (M_0_, C, and K) and adequately reflects experimental data in the range of 0 to 0.95 a_w_ for most foods [58]. The model GAB showed very good agreement with experimental data for pudding powder in the wide a_w_ range (0.11–0.90) (R^2^ ≥ 0.99; *P* and *RMSE* < 10%). The model GAB has an additional degree of freedom, K, which makes the model more versatile. The K parameter is used as a measure of the interaction between molecules in multiple layers [58]. When the K parameter is closer to one, there is almost no difference between multi-layered molecules and mono-layered molecules, indicating that the multilayered molecules behave more like liquid molecules [75]. The K parameter was calculated as 1.163 and the C parameter as 1282 for pudding powder. The magnitude of the C value is an indicator of the strong binding of water molecules in a single layer [76]. Lewicki [77] stated as a result of the mathematical analysis of the model GAB that the parameter C for the determined isotherm type should be between 5.67 < C < ∞ to correspond to the characterization of BET. The monolayer moisture content, parameter M_0_, indicates how much water is firmly adsorbed on certain areas of the top layer of the food. The M_0_ parameter helps determine the food’s chemical and physical stability as it directly affects enzyme activity, non-enzymatic browning, fat oxidation, product structure, and flavor retention. In addition, the parameter M_0_ plays a significant role in determining the optimal conditions for food storage [78], since an increase in a_w_ corresponding to an increase in the M_0_ value by 0.1 units reduces the shelf life of a food by half or even a third [52]. The M_0_ parameter values determined with the GAB model were calculated as 0.0106 kg/kg dry solids. M_0_ values indicate the ideal moisture content for the storing of pudding powder. In this study, a clear relationship was observed between the ability of the packaging materials to maintain product moisture close to M_0_ and their storage performance. Packages with lower water vapor transmission rates, such as PET/Al-foil/LDPE and PET.AlO_x_/LDPE, effectively maintained moisture levels around the monolayer region, resulting in minimal caking, color change, and sensory deterioration. Conversely, MPET/LDPE and MBOPP, which had higher permeability, allowed the product to absorb more moisture and move further away from the M_0_ region, where product instability increases. From a practical standpoint, this low M_0_ value suggests that pudding powder should be stored at relative humidities below 60% to prevent excess moisture uptake and loss of quality. Under these conditions, the risk of caking and texture changes can be minimized.

The model BET defines well the equilibrium moisture content in the range 0.110–0.500 (*P* and *RMSE* < 10%). As noted by Chirife and Iglesias [79], this model is valid for low a_w_ values. The M_0_ parameter values obtained with the BET model, which are an indicator of the sorptivity of the material, were calculated to be 0.0164 kg/kg dry solid. Literature indicates that the M_0_ value is less than 0.1 kg/kg dry solid, and this value is the highest value for food [80]. As shown in Table 2, the Peleg model also agree well with the experimental data (*P* and *RMSE* < 10%).

The comparison of the experimental data with the adsorption equilibrium moisture contents calculated with the GAB and Peleg models for pudding powder at 38 °C with a wide a_w_ range (0.11–0.9), which showed the best agreement with the experimental data (*P* and *RMSE* < 10%), is shown in Figure 1.

### 3.2. Analysis of Quality Changes in Packaged Pudding Powder Under Temperature-Dependent Accelerated Storage Conditions

#### 3.2.1. Permeability Properties of the Packaging Materials

A thorough understanding of multilayer film characteristics is of considerable practical and commercial significance, as the barrier properties of packaging materials are critical in preventing the deterioration of product quality [81]. The permeability characteristics of the packaging materials used in the study are presented in Table 1. Among all sample groups, the PET/Al-foil/LDPE exhibited the lowest OTR and WVTR values. Indeed, aluminum foil is recognized for its high moisture and oxygen barrier properties, and an intact aluminum–polyethylene laminated film is expected to exhibit oxygen and water vapor transmission rates (OTR and WVTR) below 0.1, as reported by Johansson et al. [82], which aligns well with the values obtained in our study. Although the barrier properties PET.AlO_x_/LDPE and MPET/LDPE are higher than those of the PET/Al-foil/LDPE film, the differences remain relatively small. Struller et al. [22] determined the WVTR and OTR values of a 12 μm thick standard packaging-grade PET coated with AlO_x_ as 0.56 ± 0.03 g/m^2^·day and 0.54 ± 0.05 cc/m^2^·day·atm, respectively. The barrier properties of the PET.AlO_x_/LDPE film investigated in this study are lower, likely due to the additional LDPE layer laminated to the PET. Among the film samples used in the study, the material with the highest permeability was MBOPP. Although non-metalized BOPP exhibits a lower WVTR compared to PET and PE, the reduced thickness of the MBOPP film relative to the total thickness of the MPET/LDPE film is considered to contribute to its higher WVTR values. In the literature, the WVTR and OTR values of BOPP film of 25 μm thickness are reported to be around 4–6 g/m^2^·day and 2000–2500 cc/m^2^·day·atm, respectively. It has been noted that MBOPP film, in comparison to non-metalized BOPP film, exhibits a reduction of approximately 75% in WVTR and 98.7% in OTR values [20]. In another study, it was reported that the WVTR of MBOPP 20 μm/BOPP 20 μm film varies between 1 and 2 g/m^2^·day, while its OTR was determined to be 44.6 cc/m^2^·day·atm [81]. The lower barrier values observed for the MBOPP film used in this study are likely attributable to its greater thickness.

#### 3.2.2. Moisture Content and Water Activity

Powdered food products may be significantly affected by comparatively minor changes in the moisture content of the product. Therefore, the moisture content and water activity (a_w_) are the key factors in powdered foods’ stability to control flow properties [83]. When moisture is absorbed within the powder, it reduces the powder’s ability to flow freely. The water molecules interact with the surfaces of individual particles, leading to increased stickiness. This, in turn, facilitates the formation of agglomerates or clusters by promoting inter-particle bonding. As moisture levels increase, these clusters may grow larger, eventually resulting in the fusion of particles and the formation of solid masses, or cake [84].

**Figure 2:**
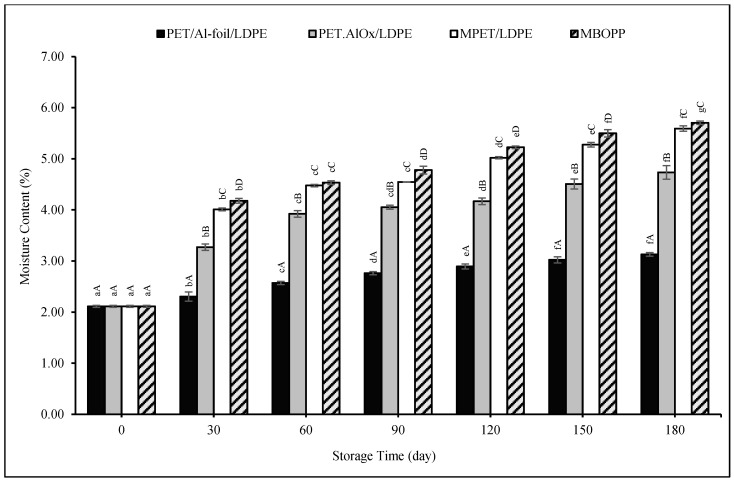
Moisture content changes of the pudding powders in different packaging materials throughout the storage period. Capital letters indicate statistically significant differences among packaging materials at each storage interval (*p* < 0.05). Lowercase letters indicate statistically significant differences over storage time within each packaging material (*p* < 0.05).

Moisture content changes in pudding powder samples packaged in different materials during the 180-day storage period are presented in Figure 2. All samples showed statistically significant changes starting from day 30, and significant increases were observed in each storage period. The initial moisture content of the samples was 2.11%, which increased gradually throughout storage. By the end of the 180-day period, the lowest moisture content (3.13%) was recorded in samples packaged in PET/Al-foil/LDPE, whereas comparatively higher values were observed in samples stored in PET.AlO_x_/LDPE (4.73%), MPET/LDPE (5.59%), and MBOPP (5.70%).

These results are consistent with the WVTR of the packaging materials, with PET/Al-foil/LDPE exhibiting the lowest WVTR, thus offering superior moisture barrier properties. Although the differences in WVTR among the materials were relatively small, PET/Al-foil/LDPE demonstrated the greatest barrier to moisture uptake. As shown in Figure 1, although the pudding powder exhibited higher moisture absorption in environments with RH above 80%, the packaging materials used provided effective protection against moisture absorption even in high-moisture conditions of 90% RH. When powdered food is packaged, it may absorb moisture from humid air present in the headspace of the package or permeating through the packaging material. The adsorption of water from the air is a time-dependent process; the water vapor permeability of the packaging material is an important property for this phenomenon.

The differences in moisture uptake behavior among the packaging materials can be further explained by the diffusion mechanism of water vapor through multilayer films. Metal foils serve as nearly absolute barriers; metal oxide coatings are amorphous and brittle; therefore, mechanical stresses generated during lamination or heat sealing can induce microcracks, increasing permeability. Moreover, AlO_x_ coatings generally possess a denser structure and stronger polymer–oxide adhesion compared to metalized or uncoated films, resulting in lower defect density and improved barrier stability [22,28]. Metalized polymer films (e.g., MPET/LDPE and MBOPP) incorporate nanometric aluminum coatings, and their barrier efficiency is highly sensitive to the continuity and microstructure of the metal layer. During vacuum deposition, the metal initially grows as isolated islands that eventually coalesce into a continuous film; if coalescence is incomplete, residual pinholes or grain-boundary voids remain and serve as fast diffusion pathways for gases and moisture [85,86]. Accordingly, the effective barrier performance correlates closely with metrics of coating uniformity such as optical density and sheet resistance and with strong metal–polymer adhesion, since these indicators reflect the formation of a continuous, defect-free metalized layer. Under high humidity, moisture plasticizes the polymer adjacent to the metal layer, causing interfacial relaxation that may widen existing discontinuities, while in foil laminates, barrier loss mainly arises from flex-induced cracks or seal-edge damage [87]. Therefore, the lowest WVTR/OTR and the smallest moisture uptake observed for PET/Al-foil/LDPE align with the expectation that an intact foil blocks diffusion almost completely; the residual transmission stems from defect-sensitive regions (seals, edges, and flex areas). In comparison, PET.AlO_x_/LDPE also exhibited very low permeability values, which can be attributed to the dense amorphous structure of the AlO_x_ coating that increases the tortuosity of diffusion pathways and limits gas and moisture permeation. Although its barrier performance is slightly lower than that of aluminum foil, the AlO_x_ layer provides an excellent balance between transparency, recyclability, and barrier stability, maintaining integrity under humid and thermal conditions. Metalized structures (MPET/LDPE and MBOPP) also show higher but still moderate transmission, consistent with nanometric metal continuity and defect density controlling transport. These results collectively explain why, under 90% RH storage, all packaging systems limited moisture gain within acceptable stability limits, with foil- and oxide-based laminates demonstrating superior long-term barrier performance, and metalized films ranking according to metal continuity, adhesion, and flex history.

Overall, the increases in moisture content were moderate, and even for MBOPP, which exhibited the highest WVTR, the moisture content remained within acceptable limits for product stability. According to the Codex Alimentarius [88], the moisture content should not exceed 7% m/m for cocoa powders and dry mixtures of cocoa and sugars. All packaging materials maintained the moisture contents of the pudding powders throughout the storage period below this threshold.

**Figure 3:**
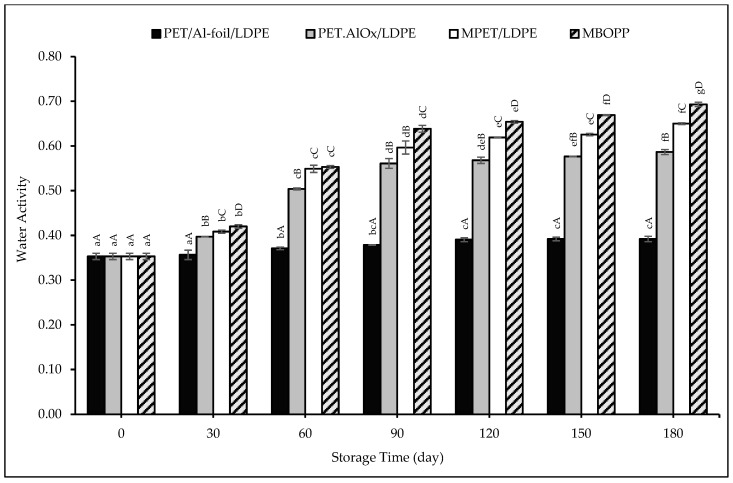
Water activity changes of the pudding powders in different packaging materials throughout the storage period. Capital letters indicate statistically significant differences among packaging materials at each storage interval (*p* < 0.05). Lowercase letters indicate statistically significant differences over storage time within each packaging material (*p* < 0.05).

The changes in the a_w_ of pudding powder samples packaged in different packaging materials with respect to storage time are presented in Figure 3. No statistically significant changes were observed in the PET/Al-foil/LDPE packaging within the first 30 days, whereas PET.AlO_x_/LDPE, MPET/LDPE, and MBOPP-packaged samples exhibited statistically significant increases throughout the storage period. A significant (*p* < 0.01) and positive (R^2^: 0.950) correlation was determined between the moisture content and the a_w_ findings. Consistent with the moisture content findings, the samples packaged in PET/Al-foil/LDPE exhibited the lowest water activity values, whereas the highest values were observed in the MBOPP-packaged samples. At the end of 180 days of storage, the a_w_ increased from an initial 0.353 to 0.392 in PET/Al-foil/LDPE samples, while it reached 0.587, 0.650 and 0.693 in PET.AlO_x_/LDPE, MPET/LDPE and MBOPP samples, respectively. These results align with the WVTR of the packaging materials. The stability of dried foods, such as baby food flour [89], kuini powder [90], tamarind pulp powder [45], papaya powder [91], and mango soy-fortified yoghurt powder [50] have been investigated in the packaging materials with different WVTR values. These studies demonstrate that both moisture absorption and the increase in a_w_ of the foods are closely correlated with the WVTR of the packaging material. Notably, packaging materials with low WVTR showed reduced moisture absorption and a smaller increase in a_w_, which is consistent with the results obtained in our study.

The a_w_ of the samples also showed a compatible behavior with the sorption isotherm given in Figure 1 and remained below the a_w_ of 0.80 during the storage period; therefore, the moisture content did not exceed 6%. Consequently, the alternative packaging materials tested can be considered suitable for pudding powder storage. Comparable studies on similar powdered products, such as powdered milkshake [92] and Palada Payasam mix, a rice-based dessert Jose et al. [93], also reported equilibrium moisture contents around 5% at water activity levels between 0.65 and 0.70.

#### 3.2.3. Caking Degree

**Figure 4:**
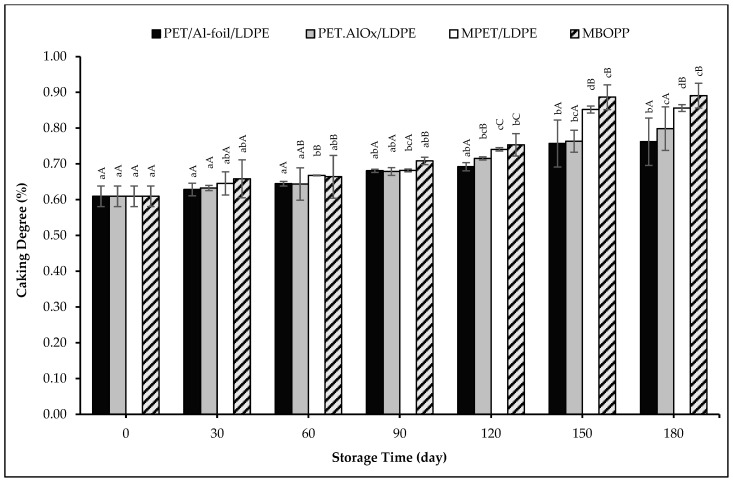
Caking degree changes of the pudding powders in different packaging materials throughout the storage period. Capital letters indicate statistically significant differences among packaging materials at each storage interval (*p* < 0.05). Lowercase letters indicate statistically significant differences over storage time within each packaging material (*p* < 0.05).

Although the initial caking degree of 0.6096% increased significantly in all packaging types over the storage period, the increases remained relatively mild, with values not exceeding 0.8909% in any sample by the end of storage, as presented in Figure 4. No visible caking was observed as confirmed by the sensory scores of the samples) and sample images. Caking showed a significant (*p* < 0.01) and negative correlation with appearance scores, with a coefficient of determination (R^2^) of −0.886. It was also confirmed that there was no change in glass transition temperature as discussed in the relevant section.

Four primary caking mechanisms in powdered foods have been identified based on their composition and storage conditions. These include caking due to amorphous sugars, moisture-induced caking, caking resulting from fat melting, and caking caused by protein adhesion [94,95]. However, the main cause of stickiness for amorphous powder is water plasticization or moisture-induced caking of particle surfaces [95]. The composition of the cocoa pudding powder analyzed in this study comprises granulated sugar (sucrose), starch, fat-reduced cocoa powder, and salt. Cocoa powder, crystalline sugar, and salt have hydrophobic properties and exhibit a lower tendency to cake compared to materials that are susceptible to plasticization by moisture [96]. Cocoa powders with fat or without fat do not undergo the humidity-caking mechanism. However, caking can be observed even in reduced-fat cocoa powders due to the melting of cocoa lipids rather than moisture effects [94,95]. Starch is a hygroscopic component and susceptible to plasticization by moisture [84,97]. Furthermore, it has been clearly shown that the presence of amorphous components capable of undergoing a glass transition can lead to caking issues [84]. An increase in the moisture content causes water to function as a plasticizer, thereby lowering the product’s glass transition temperature [84,98].

Our results align well with the moisture-induced caking mechanism, based on the moisture content increase in the samples. Caking degree showed a significant (*p* < 0.01) and positive correlation with both moisture content, with a coefficient of determination (R^2^) of 0.756. However, the increase in moisture content observed in the samples packaged with each material during the storage period did not reach levels sufficient to induce a significant rise in the caking degree or to change the glass transition temperature. In the study by Yian and Phing [90], the caking degree of kuini powder packaged in aluminum-laminated and PET pouches was determined to be 9.95% and 28.89%, respectively, starting from nearly zero under accelerated storage conditions similar to those in our study. The higher increase in caking degree values may be attributed to the greater moisture absorption of kuini powders, which rose from an initial 2.91% to 11.44% in aluminum-laminated pouches and 25% in PET pouches. However, in our study, the moisture gain was considerably lower, increasing from approximately 2% initially to a maximum of 5.57%. This marked difference in moisture uptake likely accounts for the much higher caking degree observed in the kuini powder samples, as elevated moisture levels promote particle adhesion and agglomeration.

In conclusion, although the small increases in caking measured over time were statistically significant, from a practical standpoint, such low levels are negligible and unlikely to adversely affect product quality. Even though a statistically significant increase was detected after 180 days of storage, a caking degree below 1% indicates that the powder essentially maintained its free-flowing nature, with only trace amounts of aggregation. According to industry standard tests, caking levels below approximately 10% are classified as “non-caking” [99]. Therefore, the observed caking levels are far below the threshold at which functional problems typically arise. The powder did not reach a level that could compromise its structure or form persistent lumps that would impair essential functional properties such as flowability, solubility, or mixability. Moreover, the absence of any change in glass transition temperature indicates that the amorphous components (e.g., starch) did not absorb sufficient moisture to become rubbery or sticky. Similarly, in the reduced-fat cocoa, no fat migration or melting was observed that could lead to hard caking. Overall, all tested packaging materials effectively maintained powder quality and prevented significant caking throughout the storage period. Consequently, the product’s quality attributes and consumer-perceived value were preserved, demonstrating that all packaging materials provided adequate protection against moisture-induced caking.

#### 3.2.4. Glass Transition Temperature

It has been clearly shown that the presence of amorphous components capable of undergoing a glass transition can lead to caking issues [84]. Although the cocoa powders do not have amorphous regions in their structures, caking can occur due to fat melting in cocoa powders [84]. Consequently, while cocoa powder may not exhibit a distinct glass transition temperature associated with amorphous phases, its thermal behavior, particularly the melting of lipids, plays a critical role in its physical stability.

The DSC thermogram revealed two distinct thermal transitions: an endothermic melting transition at approximately 18 °C corresponding to cocoa butter polymorph II, and a glass transition at around 100 °C associated with starch (Figure S1). It should be noted that, throughout the entire storage period, no shifts or alterations in these thermal transition temperatures were observed in any of the samples, indicating that both the lipid and starch components maintained their thermal properties and that the overall physical state of the pudding powders remained stable regardless of the packaging material used. These results also support the observation that no caking occurred in the pudding powders, indicating that the physical and thermal stability of both the starch and fat components was maintained throughout storage period.

Given the known polymorphic behavior of cocoa butter and its major triacylglycerols (TAGs), this transition temperature aligns with the melting or phase change associated with polymorph II of cocoa butter, particularly with TAGs such as POS, which melt near 19 °C [100]. The cocoa powder used in the pudding formulation contains less than 20% fat, which is within the range where residual cocoa lipids can still exhibit polymorphic transitions detectable by DSC. This is consistent with findings by Petit et al. [94], who observed caking in cocoa powders with 10–12% and 20–22% fat content upon storage at elevated temperatures (40 °C), attributing this phenomenon to the melting of cocoa lipids rather than environmental humidity. Their study confirms that even cocoa powders with moderate fat content retain sufficient lipid fractions to undergo solid-state transitions. Therefore, the transition observed at 18 °C in this study can be reasonably attributed to the polymorphic transition of cocoa butter present in the cocoa powder fraction of the pudding mix.

Starch contains amorphous and crystalline regions according to their composition. However, the T_g_ of the starch may significantly change due to its moisture content. An increase in moisture content and a_w_ causes water to function as a plasticizer, thereby lowering the T_g_ [84,98,101,102]. To clarify second transition temperature in the cacao pudding, a literature review was performed. Zeleznak and Hoseney [103] reported T_g_ values ranging between approximately 90 °C and 30 °C for wheat starch with moisture contents in the range of 13 and 20.1%. Similarly, Pérez et al. [104] determined T_g_ values between 82 °C and 35 °C for cassava starch with moisture contents in the range of 8.6 to 17.5%. Chung et al. [105] reported T_g_ values ranging from 138 °C to 35 °C for rice starch at moisture levels of 8% and 18%. Figueroa et al. [106] observed T_g_ values between 89 °C and 32.5 °C for cassava starch with moisture contents in the range of 6.5% to 16.6%. Furthermore, Liu et al. [107] reported a T_g_ of approximately 60 °C for corn starch with a moisture content of 13.7%. These studies confirm that the T_g_ of starches increases as the moisture content decreases. Considering the composition of pudding powders, primarily starch, sugars, and cocoa, other components are unlikely to account for the thermal transition observed near 100 °C. Therefore, according to the moisture contents in our samples, the thermal transition observed at approximately at 100 °C is most likely attributable to the starch phase present in the pudding system.

#### 3.2.5. Color Analysis

**Figure 5:**
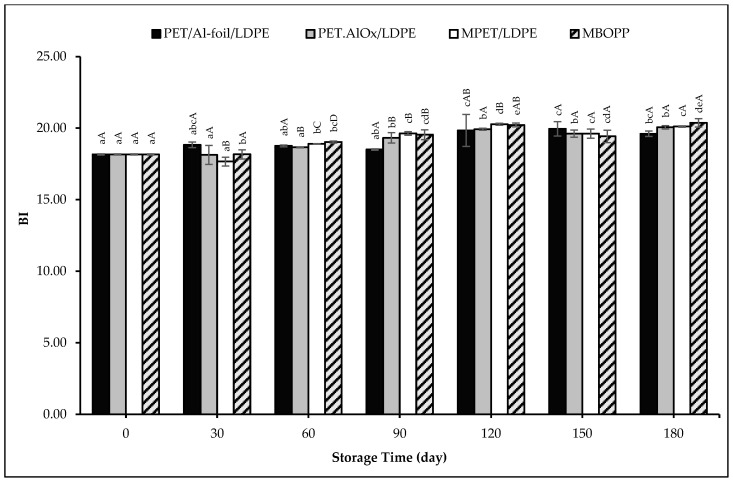
BI changes of the pudding powders in different packaging materials throughout the storage period. Capital letters indicate statistically significant differences among packaging materials at each storage interval (*p* < 0.05). Lowercase letters indicate statistically significant differences over storage time within each packaging material (*p* < 0.05).

In Figures S2–S4, the time-dependent variation of the sample groups in different packaging materials is shown with the L^*^, a^*^, and b^*^ color scales. There were only slight changes in the color indexes of the pudding powder samples throughout the storage period, L^*^ value decreased, whereas a^*^ and b^*^ values increased for all packaged samples. In a study conducted by Yian and Phing [90], it was found that both the packaging material and storage period significantly affected the preservation of L^*^, a^*^, and b^*^ color values of kuini powder, showing a similar tendency to the results obtained in our study. Greater color changes were observed in PET packaging compared to aluminum laminated pouches, which may be attributed to the higher WVTR values of PET than aluminum pouches. In another study conducted on the stability of pudding powder, polyethylene and aluminum laminated pouches were used as packaging materials, and the samples were stored at 15, 25, 35, and 45 °C. The results showed that the changes in L^*^, a^*^, and b^*^ values during the storage period were similar to those observed in our study, being affected by both the storage period and the WVTR of the packaging materials [108]. A very slight darkening was observed in all packaged samples by the end of the storage period, as shown in Figure S5. The observed darkening corresponds with a slight increase in the Browning Index (BI). As shown in Figure 5, the initial BI value of 18.16 increased slightly over the storage period, reaching values between 19.60 and 20.37 for all packaging materials by the end of the 180-day storage period. The lowest BI was observed for the PET/Al-foil/LDPE sample, while the highest was for the MBOPP sample. Due to the low a_w_ of powdered products, these are susceptible to non-enzymatic darkening reactions [109]. But the non-enzymatic chemical reactions are not seen in powder pudding samples in our study owing to their composition. However, moisture absorption and caking are the main causes of the darkening of powdered food products, since they reflect off the light. As also indicated by our moisture and caking results, the minimal changes observed suggest that the extent of darkening was also very limited. The BI values were significantly (*p* < 0.01) and positively correlated with moisture content, the a_w_ and caking degree findings with coefficients of determination (R^2^) of 0.643, 0.723 and 0.758, respectively.

#### 3.2.6. Sensory Analysis

**Figure 6:**
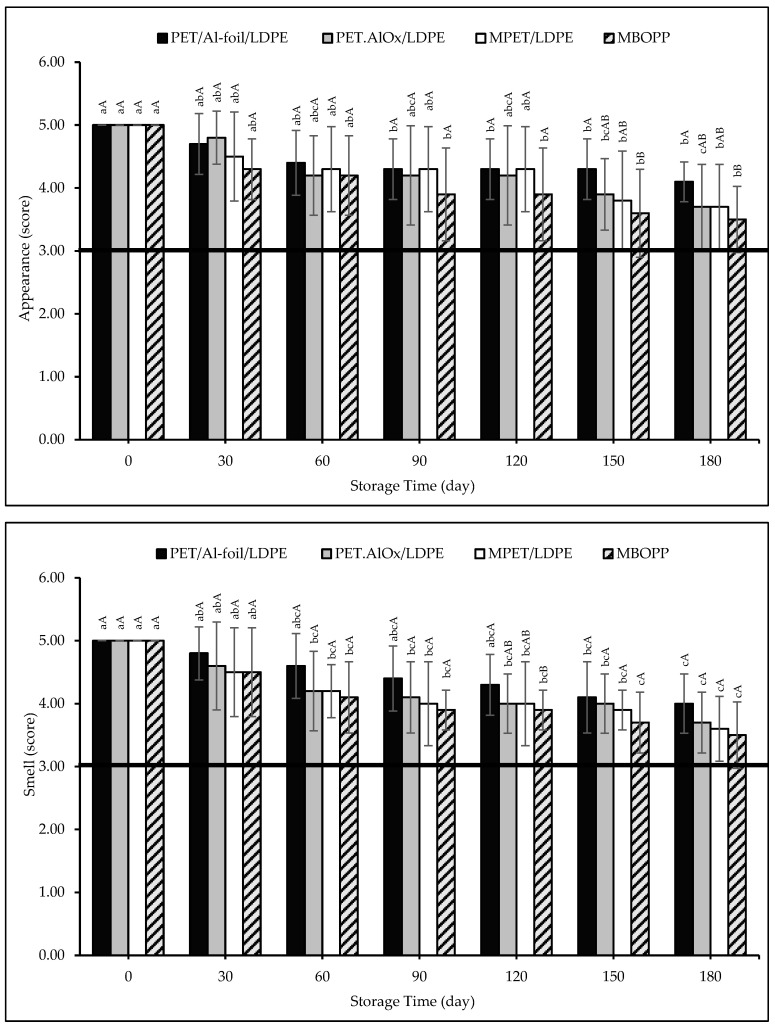

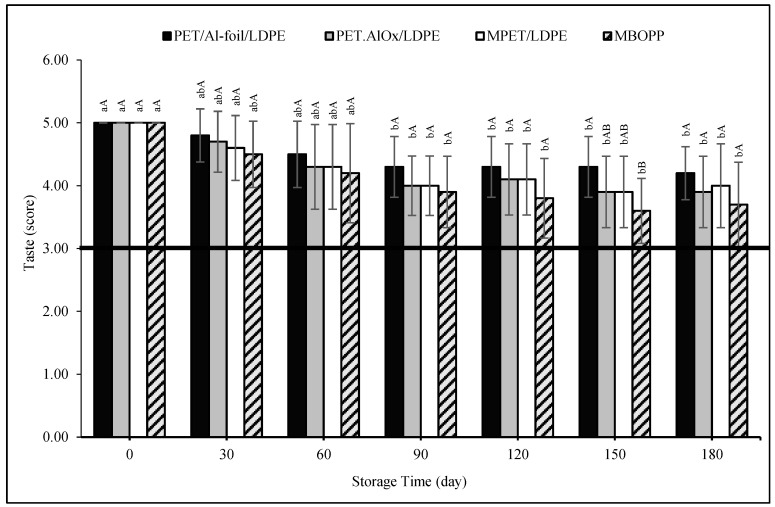
Appearance, smell and taste scores of the pudding powders in different packaging materials throughout the storage period. Capital letters indicate statistically significant differences among packaging materials at each storage interval (*p* < 0.05). Lowercase letters indicate statistically significant differences over storage time within each packaging material (*p* < 0.05).

The sensory evaluation scores are illustrated in Figure 6. In the appearance analysis, the factors such as uniformity, caking, and color change were assessed. While the loss of the characteristic cocoa pudding scent was evaluated in the smell analysis, the loss of the characteristic cocoa pudding aroma was focused on in the taste analysis. Overall, a slight decline in appearance, smell, and taste scores was observed over the 180-day storage period. This trend was more pronounced in samples packaged in MBOPP, while the PET/Al-foil/LDPE packaging consistently preserved the sensory attributes. No caking was observed in any of the samples, and minor darkening of the pudding powders was primarily responsible for the reduction in appearance scores. Appearance scores were significantly (*p* < 0.01) and negatively correlated with caking and BI values (R^2^ = −0.888 and 0.756, respectively), indicating that color changes rather than physical clumping affected visual perception. In terms of smell, slight decreases were observed from day 30 onwards, consistent with differences in OTR of the packaging materials. PET/Al-foil/LDPE maintained the highest smell scores throughout storage, suggesting that its superior barrier properties minimized aroma loss. No foreign or undesirable odors were detected. Taste scores followed a similar pattern: initial declines were observed for all samples, but PET/Al-foil/LDPE maintained stable scores after day 30, while other materials experienced further decline by day 90. Perceived bitterness, cocoa flavor, and sweetness remained comparable across all samples, reflecting the absence of significant chemical degradation such as protein denaturation or lipid oxidation, and no foreign tastes developed. None of the samples fell below the sensory analysis threshold value of 3.0 for any sensory attribute, indicating that all packaging materials can be used to preserve the sensory attributes of the pudding powder samples, such as appearance, odor, and taste, during the 180-day storage period, even under accelerated storage conditions. In a study investigating the effect of packaging materials on the sensory properties of dried durian fruit leather, the samples were packaged in four different materials: laminated aluminum foil, high density polyethylene, low density polyethylene, and polypropylene, and stored at 27 °C for 12 weeks. According to the sensory evaluation results, the highest scores were obtained for the samples packaged in laminated aluminum foil due to its higher barrier properties, and all packaged samples remained sensory acceptable by the end of the storage period, in accordance with our results [110].

### 3.3. Acceptable Storage Period of the Pudding Powder

The acceptable storage period of packaged samples is influenced by multiple factors, including the water vapor permeability (WVTR) of the packaging material, the sorption characteristics of the product, and water activity gradients induced by storage temperature and relative humidity [41]. In this study, the shelf life of the pudding powder was estimated by applying Equation (6), which incorporates both the WVTR values of each packaging type and the GAB model parameters derived from sorption isotherm data. The change in moisture content is related to storage conditions, the value of water activity in the package’s headspace, and the permeability of the package [41,71]. After 180 days of storage under accelerated conditions (38 °C, 90% RH), the final moisture contents of the samples packaged in PET/Al-foil/LDPE, PET.AlO_x_/LDPE, MPET/LDPE, and MBOPP at the end of storage period were 3.13, 4.73, 5.59, and 5.70%, respectively. Based on these moisture uptake values, corresponding GAB-modelled sorption behavior and water vapor permeability coefficient of packaging materials, the predicted shelf life was estimated as 800 days for PET/Al-foil/LDPE, 578 days for PET.AlO_x_/LDPE, 408 days for MPET/LDPE, and 229 days for MBOPP. Due to its higher-water vapor permeability, the pudding powder packed with MBOPP had the shortest shelf life. These values are in accordance with WVTRs’ packaging materials.

The shelf life predictions obtained in this study correlate strongly with the respective WVTR values of the packaging materials, supporting the conclusion that improved barrier properties contribute to longer storage stability. However, a key limitation of this approach is that the shelf life estimates are based on data obtained during 180 days of accelerated storage period. Furthermore, since this modelling approach estimates shelf life using only a single criterion (moisture content/water activity), the predicted shelf life is valid only in relation to moisture-related quality changes. Considering these limitations, the shelf life values reported in this study should be interpreted as comparative indicators among the tested packaging materials rather than absolute measures.

## 4. Conclusions

Although the unpackaged pudding powder exhibited high moisture absorption in environments with relative humidity above 80%, all tested packaging materials effectively limited moisture uptake, preserving caking, color, browning, glass transition temperature, and sensory attributes. In terms of moisture content, water activity, and caking degree, conventional PET/Al-foil/LDPE demonstrated the best performance, followed by PET.AlO_x_/LDPE, MPET/LDPE, and MBOPP. Sensory analysis scores followed a similar ranking, though all samples remained sensorially acceptable throughout the 180-day storage period. Estimated acceptable storage periods under accelerated conditions ranged from approximately 229 to 800 days depending on packaging material.

From an industrial perspective, MBOPP packaging may be suitable for short-term storage and fast product turnover due to its recyclability and mono-layered structure, despite a shorter shelf life. PET.AlO_x_/LDPE and MPET/LDPE offer a balance between sustainability and shelf-life performance, suitable for products requiring 1–2 years of storage. For extended storage exceeding 2 years, PET/Al-foil/LDPE provides superior barrier properties, though it involves more layers and may be less easily recyclable.

For stable and durable food products such as pudding powders, packaging materials should be selected based on their barrier performance, moisture protection, and ability to preserve sensory and physical properties. Materials such as PET/Al-foil/LDPE provide superior protection for long-term storage, while PET.AlO_x_/LDPE and MPET/LDPE offer a good balance between shelf-life and sustainability. Mono-layer, recyclable materials like MBOPP may be suitable for short-term storage or fast product turnover, highlighting the trade-off between durability and environmental considerations.

These findings highlight the importance of designing packaging strategies in line with product characteristics and shelf-life requirements, taking into account the trade-off between barrier performance and sustainability. Choosing fewer layers and easily recyclable materials can support sustainable packaging initiatives, provided that product stability and sensory quality are maintained.

Despite providing valuable insights into the relationship between packaging structure and the storage stability of cocoa pudding powder, this study has certain limitations. The storage tests were conducted under accelerated laboratory conditions, which may not fully reflect real market environments. Therefore, future studies should evaluate the product performance under actual storage and distribution conditions to validate these findings. Additionally, further research should incorporate life cycle assessment and recycling cost analyses to optimize the balance between barrier performance and sustainability in packaging design.

## Figures and Tables

**Table 1:** Barrier properties of the packaging materials and predicted acceptable storage period of powder pudding.

Film Type	Explanation	OTR (cc/m^2^·Day·Atm)	WVTR (g/m^2^·Day)	Acceptable Storage Period (Days)
PET/Al-foil/LDPE (Control)	12 µm PET (Polyethylene terephthalate)/ 9 µm Al-Foil (Aluminum foil/60 µm LDPE (Low density polyethylene)	0.026 ± 0.002	0.049 ± 0.053	800.32
PET.AlO_x_/LDPE	12 µm PET.AlO_x_ (AlO_x_-coated PET)/ 50 µm LDPE	0.130 ± 0.000	0.190 ± 0.020	577.92
MPET/LDPE	12 µm MPET (Metallized polyethylene terephthalate)/50 µm LDPE	0.335 ± 0.134	0.356 ± 0.035	407.58
MBOPP	30 µm MBOPP (Metallized biaxially oriented polypropylene)	12.190 ± 1.965	0.650 ± 0.050	229.26

**Table 2:** Sorption model equations and estimated sorption model parameters of powder pudding, R^2^ and RMSE (%) values.

Model		Parameters	
Name	Equation		
BET * [57]	$M\;=\frac{M_{0}Ca_{w}}{\left[\left(1-a_{w}\right)+\left(C-1\right)\left(1-a_{w}\right)a_{w}\right]}$	M_0_	0.0164
		C	13.150
		P (%)	5.09
		RMSE (%)	6.55
		R^2^	0.977
GAB [58]	$M\;=\frac{M_{0}Cka_{w}}{\left[\left(1-{\mathrm{ka}}_{w}\right)\left(1-{\mathrm{ka}}_{w}+Cka_{w}\right)\right]}$	M_0_	0.0106
		C	1282
		K	1.163
		P (%)	3.66
		RMSE (%)	0.045
		R^2^	0.9953
Halsey [59]	$a_{w}=e^{\left[\left(-k\right)/M^{n}\right]}$	k	0.111
		n	0.353
		P (%)	10.89
		RMSE (%)	0.123
		R^2^	0.972
Iglesias-Chirife [60]	$a_{w}=\frac{\left(ln\left[M+{{(M}^{2}+M_{0.5})}^{0.5}\right]-c\right)}{k}$	k	36.798
		c	−1.260
		M_0.5_	0.0311
		P (%)	1.524
		RMSE (%)	0.027
		R^2^	0.856
Henderson [61]	$1-a_{w}=e^{\left(-kM^{n}\right)}$	k	2.316
		n	0.184
		P (%)	11.95
		RMSE (%)	0.004
		R^2^	0.964
Peleg [62]	$M=k_{1}{a_{w}}^{n_{1}}+k_{2}{a_{w}}^{n_{2}}$	k_1_	0.0514
		n_1_	0.7392
		k_2_	18.598
		n_2_	24.407
		P (%)	4.277
		RMSE (%)	0.0015
		R^2^	0.999

M_0_, C, K, c, k_1_, k_2_, n, n_1_, and n_2_ are the constants in the sorption isotherm models; a_w_, is the water activity; M is the equilibrium moisture content (dry base); M_0.5_ is the moisture content (dry base) when a_w_ is 0.5; * a_w_ range is 0.11–0.5 for BET equations. First five data were used for equation.

## Data Availability

The data that support the findings of this study are available on request from the corresponding author.

## Supplementary Materials

The following supporting information can be downloaded at: https://www.mdpi.com/article/10.3390/foods14223806/s1, Figure S1: DSC curves of powdered puddings packaged with MBOPP at (**a**) day 0, (**b**) 3 months storage, (**c**) 6 months storage; Figure S2: Changes in *L*^*^ index of the pudding powders in different packaging materials throughout the storage period. Capital letters indicate statistically significant differences among packaging materials at each storage interval (*p* < 0.05). Lowercase letters indicate statistically significant differences over storage time within each packaging material (*p* < 0.05); Figure S3: Changes in *a*^*^ index of the pudding powders in different packaging materials throughout the storage period. Capital letters indicate statistically significant differences among packaging materials at each storage interval (*p* < 0.05). Lowercase letters indicate statistically significant differences over storage time within each packaging material (*p* < 0.05); Figure S4: Changes in *b*^*^ index of the pudding powders in different packaging materials throughout the storage period. Capital letters indicate statistically significant differences among packaging materials at each storage interval (*p* < 0.05). Lowercase letters indicate statistically significant differences over storage time within each packaging material (*p* < 0.05); Figure S5: Appearances of pudding powder samples (**a**) on initial day; after 6-months storage in (**b**) PET/Al-foil/LDPE, (**c**) MPET/LDPE, (**d**) PET.AlOx/LDPE, (**e**)MBOPP; Table S1: The criteria for sensory analysis evaluation.
