# Supplementary material for: Sustainable Alternatives in Multilayer Packaging: Storage Stability of Pudding Powder Under Accelerated Storage Conditions
Source: Foods. 2025 Nov 7;14(22):3806. doi: 10.3390/foods14223806 (PMC12650960; doi:10.3390/foods14223806)
Supplement: Supplementary file 1 [file foods-14-03806-s001.zip › foods-3933297-supplementary.pdf]

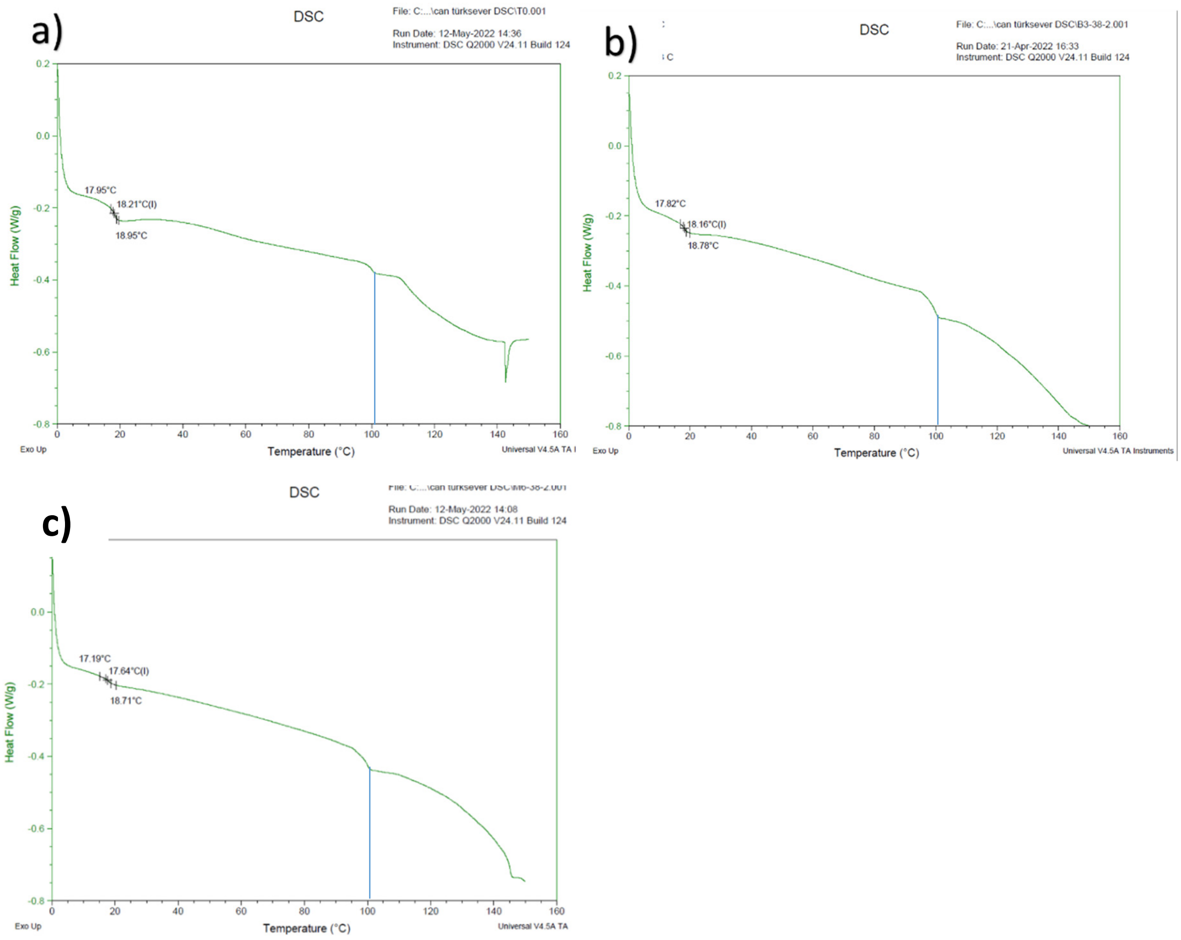

Figure S1.DSC curves of powdered puddings packaged with MBOPP at (**a**) day 0, (**b**) 3 months storage, (**c**) 6 months storage.

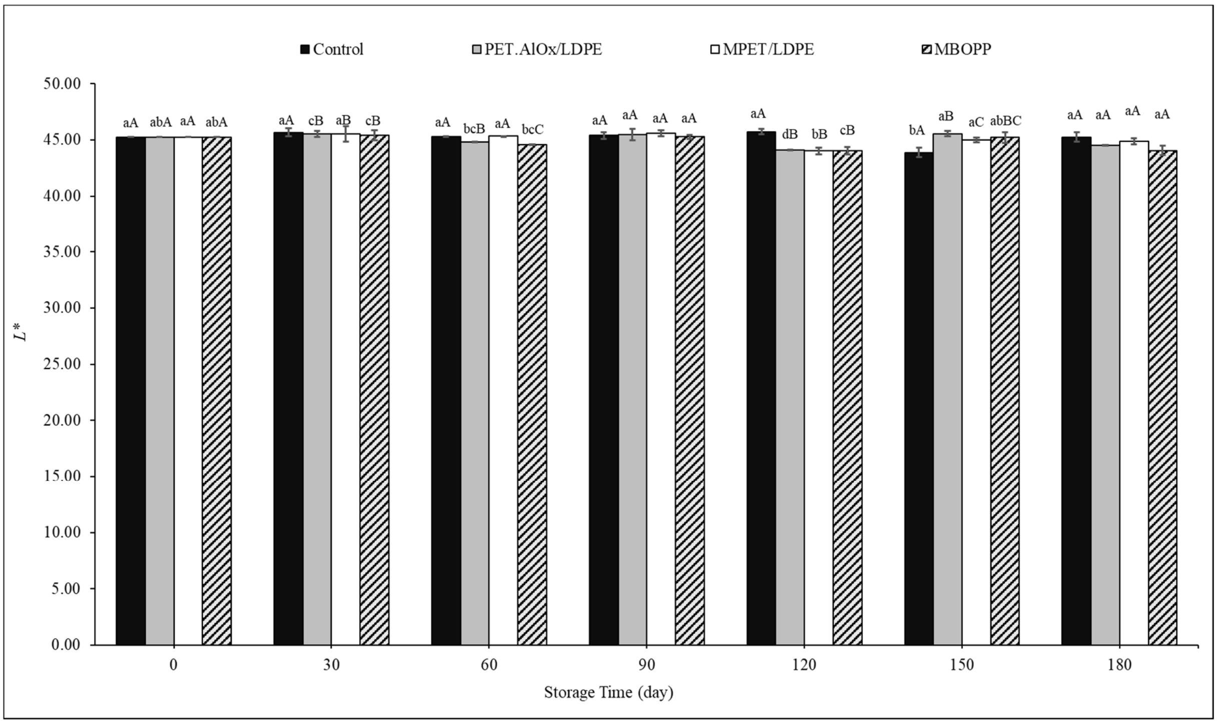

Figure S2. Changes in L^* index of the pudding powders in different packaging materials throughout the storage period. Capital letters indicate statistically significant differences among packaging materials at each storage interval ($p < 0.05$). Lowercase letters indicate statistically significant differences over storage time within each packaging material ($p < 0.05$).

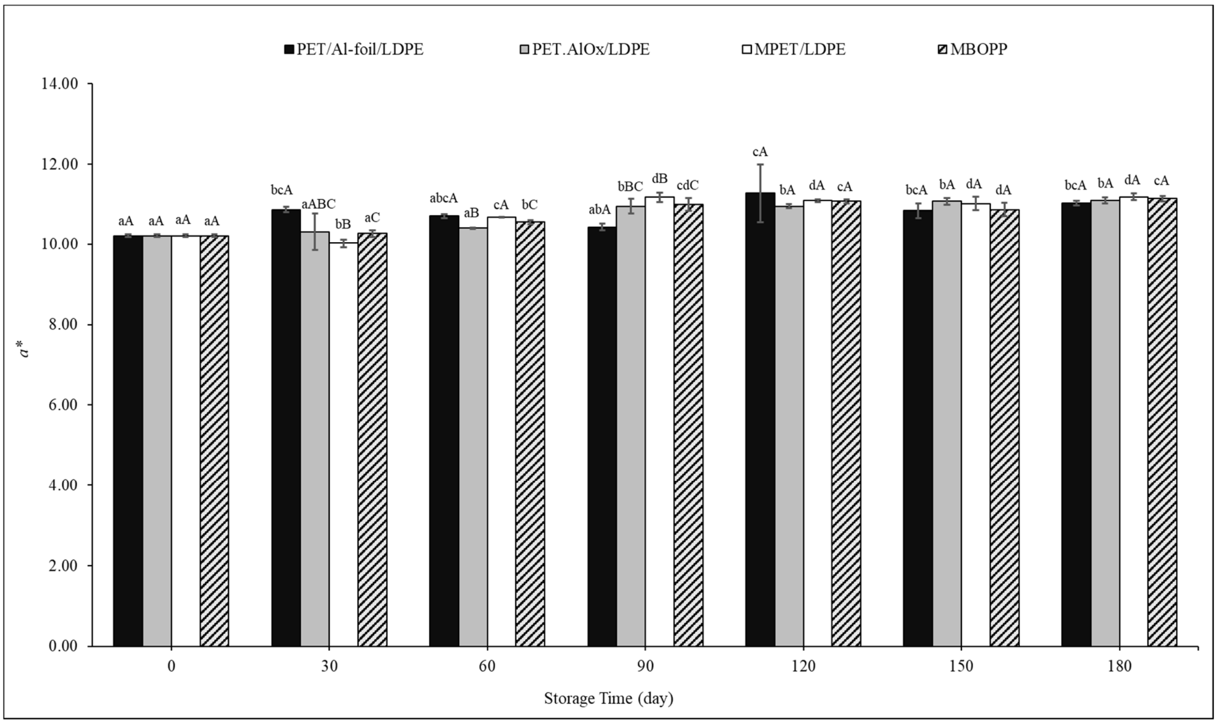

Figure S3. Changes in a^* index of the pudding powders in different packaging materials throughout the storage period. Capital letters indicate statistically significant differences among packaging materials at each storage interval ($p < 0.05$). Lowercase letters indicate statistically significant differences over storage time within each packaging material ($p < 0.05$).

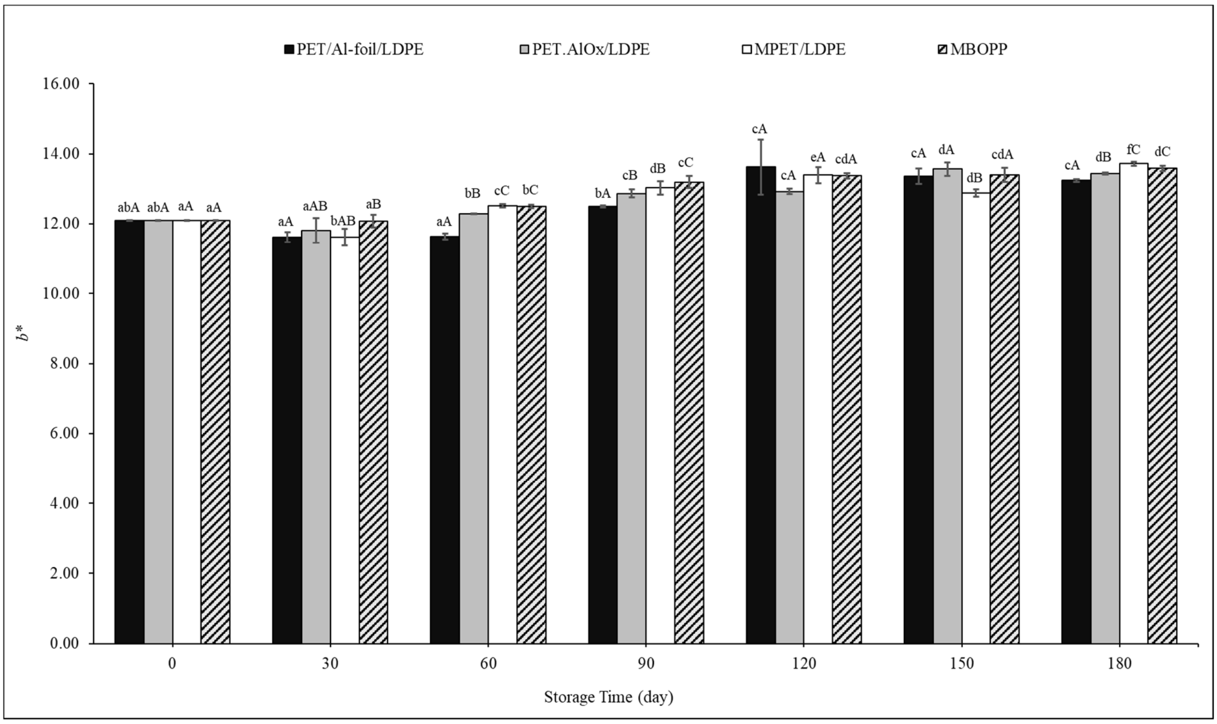

Figure S4. Changes in b^* index of the pudding powders in different packaging materials throughout the storage period. Capital letters indicate statistically significant differences among packaging materials at each storage interval ($p < 0.05$). Lowercase letters indicate statistically significant differences over storage time within each packaging material ($p < 0.05$).

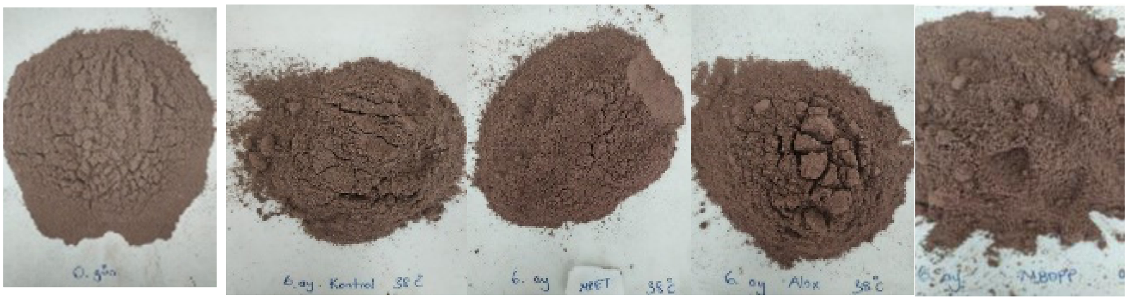

Figure S5. Appearances of pudding powder samples (**a**) on initial day; after 6-months storage in (**b**) PET/Al-foil/LDPE, (**c**) MPET/LDPE, (**d**) PET.AlOx/LDPE, (**e**) MBOPP.

Table S1. The criteria for sensory analysis evaluation.

Appearance	
**Score**	**Definition**
5	Floury, homogeneous structure, characteristic pudding powder colour
4	Slight caking , very slight darkening of the colour
3	Local caking, slight darkening of the colour
2	Heterogeneity structure, clear caking, clear darkening of the color
1	Large lumpy appearance, coarse and petrified particles, very dark colour
**Smell**	
**Score**	**Definition**
5	Dominant characteristic cocoa smell
4	Slight reduction in cocoa smell
3	Still cocoa smell
2	No cocoa smell
1	No cocoa smell and foreign smell
**Taste**	
**Score**	**Definition**
5	Characteristic cocoa pudding taste
4	Slight decrease in cocoa pudding taste
3	Reduced taste of cocoa pudding
2	No characteristic cocoa pudding taste
1	No characteristic cocoa pudding taste, foreign taste is perceived
